# Body Surface Area Indexing Attenuates Apparent Early eGFR Decline After Sleeve Gastrectomy: A Retrospective Cohort Study

**DOI:** 10.3390/jcm15083001

**Published:** 2026-04-15

**Authors:** Emre Cankaya, Hakan Babaoglu, Feyza Bayrakdar Çağlayan, Semahat Karahisar Sirali, Oktay Banli, Mehmet Emin Demir, Fatih Dede

**Affiliations:** 1 Department of Nephrology, Ankara Bilkent City Hospital, Ankara 06800, Turkey; 2 Department of Rheumatology, Ankara Bilkent City Hospital, Ankara 06800, Turkey; hakanbabaoglu@gmail.com; 3 Department of Nephrology, Faculty of Medicine, Yuksek Ihtisas University, Ankara 06520, Turkey; 4 Department of Nephrology, Faculty of Medicine, Ufuk Universiy, Ankara 06520, Turkey; 5 Obesity Surgery Ankara, Ankara 06680, Turkey; 6 Department of Nephrology, Faculty of Medicine, Atilim University, Ankara 06830, Turkey

**Keywords:** bariatric surgery, sleeve gastrectomy, body surface area, glomerular filtration rate, kidney function tests, renal hyperfiltration, absolute eGFR

## Abstract

**Background**: Early postoperative changes in creatinine-based estimated glomerular filtration rate (eGFR) after bariatric surgery can be misread as a kidney injury. During rapid weight loss, indexing eGFR to a fixed body surface area (BSA) of 1.73 m^2^ may alter apparent trajectories. We compared absolute (mL/min) and BSA-indexed (mL/min/1.73 m^2^) eGFR changes after sleeve gastrectomy, stratified by baseline glomerular hyperfiltration (GH). **Methods**: In this retrospective cohort of 145 adults undergoing laparoscopic sleeve gastrectomy, serum creatinine was obtained at baseline (≤30 days pre-op) and 3 months (post-op days 75–105). Indexed eGFR was calculated with the Chronic Kidney Disease Epidemiology Collaboration (CKD-EPI) 2021 creatinine equation; BSA with the Mosteller formula; and absolute eGFR as indexed eGFR × (BSA/1.73). GH was defined as indexed eGFR ≥ 120 mL/min/1.73 m^2^. A REML mixed-effects model (Group, Time, Group × Time) with patient-cluster bootstrap inference was used. An age-adjusted sensitivity model including Age and Age × Time was also fitted. **Results**: Fifty-four participants (37%) met the GH criteria. Absolute eGFR declined by −26.6 mL/min in GH versus −17.3 mL/min in non-GH (difference-in-differences [DiD] −9.3 mL/min; 95% CI −13.9 to −4.7; *p* < 0.001). The indexed eGFR changes were smaller (−4.2 vs. −0.5 mL/min/1.73 m^2^; DiD −3.7; 95% CI −7.3 to −0.03; *p* = 0.048; bootstrap *p*_sign = 0.052). In the age-adjusted sensitivity model, the Group × Time interaction for absolute eGFR attenuated but remained statistically significant (−6.57 mL/min; 95% CI, −13.09 to −0.06; *p* = 0.048), whereas the corresponding interaction for indexed eGFR was attenuated and no longer statistically significant (−3.99 mL/min/1.73 m^2^; 95% CI −9.15 to 1.16; *p* = 0.129). **Conclusions**: Within three months after sleeve gastrectomy, participants with higher baseline indexed filtration showed a larger decline in absolute eGFR but only a small change in indexed eGFR. These results show that early postoperative creatinine-based eGFR trajectories are scale dependent and should be interpreted cautiously during rapid weight loss. Because postoperative acute kidney injury (AKI) was not adjudicated and direct kidney function markers were unavailable, this study does not distinguish physiological hemodynamic change from structural kidney injury. Reporting both absolute and indexed eGFR may improve early postoperative interpretation and help align dosing decisions with rapid changes in body size.

## 1. Introduction

Obesity induces sustained changes in renal hemodynamics. Enhanced tubular sodium reabsorption, increased sympathetic activity, and upregulation of the renin–angiotensin–aldosterone system (RAAS) collectively raise intraglomerular pressure and promote glomerular hyperfiltration (GH) [1]. Independent of hypertension and diabetes, persistent GH accelerates glomerular injury by increasing transcapillary pressure and imposing mechanical stress on podocytes [1]. Bariatric surgery reduces these hemodynamic drivers through substantial weight loss, improved insulin sensitivity, and suppression of neurohormonal tone, and has been associated with improved renal risk profiles and long-term outcomes [2,3,4]. Interpreting early postoperative changes in creatinine-based eGFR, however, remains difficult. During rapid weight loss, shifts in body size (BSA) and creatinine generation can alter estimated glomerular filtration rate (eGFR) estimates, which complicates the distinction between hemodynamic readjustment, structural injury, and measurement artifacts.

The central methodological issue involves indexing eGFR to a fixed BSA of 1.73 m^2^. Because indexed eGFR = absolute eGFR × (1.73/BSA), a rapid postoperative reduction in BSA can inflate the indexed value at any given absolute filtration rate, creating a size-dependent artifact. Indexing can attenuate apparent declines or mask true absolute reductions. It can also complicate drug-dosing decisions when thresholds are applied using mismatched units [5,6,7,8]. This concern is particularly pronounced when a high baseline BSA rapidly approaches the population mean after surgery.

Recent guidance explicitly addresses this limitation. Kidney Disease: Improving Global Outcomes (KDIGO) 2024 recommends using non-indexed eGFR for drug dosing in individuals at extremes of body size. The Food and Drug Administration (FDA) March 2024 Final Guidance similarly advises using absolute eGFR (mL/min) rather than BSA-indexed values to guide drug dosing in patients with substantially altered body size in order to avoid unit-mismatch errors [9,10]. In line with these standards, we report eGFR on both absolute (mL/min) and indexed (mL/min/1.73 m^2^) scales and interpret early postoperative changes with attention to potential indexing artifacts, particularly at body-size extremes [9,10]. Methodological and clinical reports further indicate that de-indexing eGFR can reclassify chronic kidney disease (CKD) stages and dosing categories in obesity, supporting routine reporting of both absolute and indexed values [11,12,13,14]. Studies using measured glomerular filtration rate (GFR) before and after bariatric surgery have also described early postoperative declines in filtration, particularly among patients with higher baseline filtration, and provide physiologic context for interpreting early creatinine-based eGFR changes after surgery [15,16,17]. Despite this evolving literature, many bariatric reports and routine clinical datasets still emphasize indexed eGFR, and the magnitude and direction of divergence between indexed and absolute trajectories in the early postoperative period—particularly among those starting from hyperfiltration—remain incompletely characterized.

Studies using measured glomerular filtration rate (mGFR) provide physiological context. Friedman et al. (2014) observed a mean postoperative mGFR decline of approximately 17 mL/min using iohexol clearance; Lieske et al. (2014) similarly reported a decrease in mGFR during the first postoperative year in women; and Clerté et al. found that mGFR decreased significantly among patients with baseline hyperfiltration [15,16,17]. Together, these studies suggest that early postoperative declines may reflect hemodynamic readjustment in at least some patients. However, mechanistic inference from creatinine-based observational analyses remains limited without measured GFR or cystatin C [15,16,17,18,19,20]. Mechanistically, this postoperative decline in filtration pressure aligns conceptually with the renoprotective “dip” observed with sodium–glucose cotransporter-2 (SGLT2) inhibitors [18,19,20].

Given these uncertainties, it remains unclear whether early postoperative declines in eGFR represent physiological normalization or true renal injury, particularly when both absolute and indexed measures are evaluated concurrently. This distinction has direct clinical relevance, as misclassification could lead to misinterpretation of kidney function trajectories or inappropriate medication dose adjustments during early postoperative follow-up.

Accordingly, this study aimed to compare absolute (mL/min) and indexed (mL/min/1.73 m^2^) eGFR trajectories in adults undergoing laparoscopic sleeve gastrectomy using baseline (Month 0) and routine Month 3 follow-up measurements. We hypothesized that patients with baseline glomerular hyperfiltration (GH), defined pragmatically using indexed eGFR for comparability with prior outcome literature, would demonstrate a larger decline in absolute eGFR but a smaller decline on the indexed scale. Such a pattern would be consistent with early postoperative hemodynamic change and potential indexing-related interpretive artifacts during rapid weight loss.

## 2. Materials and Methods

We conducted a retrospective observational cohort study of 145 consecutive adults who underwent laparoscopic sleeve gastrectomy at a tertiary metabolic surgery center between 1 September 2023 and 1 May 2024. Eligible participants were adults aged 18–60 years with a body mass index (BMI) > 35 kg/m^2^ who had completed preoperative evaluation and received sleeve gastrectomy.

We defined baseline (Month 0) serum creatinine as the value measured within 30 days before surgery (closest to the surgery date), and Month 3 serum creatinine as the value obtained during postoperative days 75–105 (closest to day 90). The analytic cohort included patients with paired Month 0 and Month 3 creatinine values within these prespecified windows. As paired values were available for all 145 patients, we performed a complete-case analysis and did not impute missing data. Because the retrospective dataset did not contain serial perioperative creatinine measurements and urine-output records required for standardized AKI classification, postoperative AKI events could not be adjudicated formally.

Serum creatinine was measured using an IDMS-aligned enzymatic assay. We calculated estimated glomerular filtration rate (eGFR) using the CKD-EPI 2021 creatinine equation, which reports indexed eGFR in mL/min/1.73 m^2^ [21]. Measured GFR and cystatin C were not available in this retrospective cohort. We computed the body surface area (BSA) using the Mosteller formula and derived the absolute eGFR (mL/min) by de-indexing indexed eGFR as follows: Absolute eGFR = Indexed eGFR × (BSA/1.73) [22]. Time-specific BSA was calculated using contemporaneous weight (Month 0 and Month 3) and baseline height for the de-indexing step. Consistent with KDIGO 2024 recommendations and FDA guidance, we report absolute (non-indexed) eGFR to support drug-dosing decisions in individuals at body-size extremes or with substantially altered body size [9,10,23]. We defined baseline glomerular hyperfiltration (GH) as indexed eGFR ≥ 120 mL/min/1.73 m^2^, consistent with prior outcome studies in type 2 diabetes and hyperfiltration [24]. Because GH was defined using an indexed threshold in an analysis focused on indexing artifacts, we treated GH subgrouping as a pragmatic analytic stratification based on higher baseline indexed filtration rather than as a definitive diagnostic label. Accordingly, absolute eGFR was designated as the primary outcome and indexed eGFR analyses were secondary.

We assessed the normality of baseline continuous variables using the Shapiro–Wilk test [25]. We summarized continuous variables as the mean ± standard deviation (SD). When distributions were non-normal, we reported the median (interquartile range). Based on those results, we compared baseline characteristics between groups using Welch’s *t*-test for parametric data or the Mann–Whitney U test for non-parametric data. For longitudinal analyses, we used a restricted maximum-likelihood (REML) linear mixed-effects model [26]. The primary longitudinal analysis included fixed effects for Group (Hyperfiltration vs. Non-hyperfiltration), Time (Month 0 vs. Month 3), and the Group × Time interaction, with a random intercept for each participant. Because the groups differed substantially in age at baseline, we used a targeted age-adjusted sensitivity model that also included mean-centered age and an Age × Time interaction term as the primary confounding analysis.

We defined sign conventions as follows: between-group contrasts represent hyperfiltration minus non-hyperfiltration, and within-group change (Δ) represents Month 3 minus Month 0. The between-group difference in change (Group × Time interaction) was defined as ΔHyper − ΔNon (labeled “DiD”). Given the single-cohort design, we used the Group × Time interaction to evaluate heterogeneity in postoperative change across physiological subgroups rather than to estimate an effect relative to a non-surgical control arm. We examined residual distributions using the Shapiro–Wilk [25] and Anderson–Darling tests [27]. Because we observed deviations from normality (Supplementary Table S1), we assessed model robustness using patient-cluster bootstrap resampling (B = 2000) to obtain percentile-based 95% confidence intervals (CIs) and a sign-probability *p*-value [28]. The two-sided sign-probability *p*-value was defined as *p*_sign = 2 × min {Pr(β_boot > 0), Pr(β_boot < 0)}. We also assessed distributional sensitivity using Box–Cox transformations [29]. Finally, we computed relative DiD (difference in % change from Month 0) from model-estimated marginal means as [(ΔHyper/MeanHyper,Month0) − (ΔNon/MeanNon,Month0)] × 100 (percentage points). 

To aid clinical interpretation, we back-transformed Box–Cox-transformed estimates to the original scale using smearing retransformation based on 10,000 simulations (Supplementary Table S2) [30]. All tests were two-sided with α = 0.05.

All analyses were performed using Python (version 3.14; Pyton Software Foundation, Wilmington, DE, USA) with the statsmodels package.

We conducted this study in accordance with the Declaration of Helsinki. The study protocol was approved by the Yuksek İhtisas University Health Sciences Research Ethics Committee (Approval No. 365, Meeting No. 20, 17 February 2026). Due to the retrospective nature of the study, the requirement for informed consent was waived by the ethics committee. All data were de-identified and obtained from routine clinical follow-up; therefore, individual informed consent was waived in compliance with local regulations.

During the preparation of this manuscript, the authors used ChatGPT (version GPT-5.3, OpenAI, San Francisco, CA, USA) and Gemini (version 3.1 Pro, Google LLC, Mountain View, CA, USA) for language editing and grammatical refinement.

## 3. Results

A total of 145 adults who underwent laparoscopic sleeve gastrectomy were included; 54 (37%) met the GH criteria (indexed eGFR ≥ 120 mL/min/1.73 m^2^). Baseline demographic and biochemical variables are summarized in Table 1.

The postoperative anthropometric changes (Month 0 to Month 3) are summarized in Table 2. Mean BSA decreased from 2.42 ± 0.29 to 2.12 ± 0.24 m^2^ in the GH group (Δ = −0.30 ± 0.08 m^2^) and from 2.30 ± 0.21 to 2.04 ± 0.17 m^2^ in the non-GH group (Δ = −0.27 ± 0.07 m^2^). Participants with GH were younger (26.6 ± 4.2 vs. 41.3 ± 8.5 years; *p* < 0.001) and had higher body weight and a larger body surface area; BMI was similar between groups. Baseline serum creatinine was lower in the GH group (0.67 ± 0.09 mg/dL) than in the non-GH group (0.74 ± 0.11 mg/dL), while both indexed and absolute eGFR values were higher (125.35 ± 4.45 vs. 107.43 ± 10.16 mL/min/1.73 m^2^ and 175.21 ± 23.11 vs. 143.12 ± 18.77 mL/min; all *p* < 0.001). 

Table 3 and Table 4 present model-derived eGFR means and between-group contrasts. At 3 months postoperatively, mean absolute eGFR decreased from 175.2 to 148.7 mL/min in the GH group and from 143.1 to 125.9 mL/min in the non-GH group, corresponding to within-group changes of −26.6 mL/min and −17.3 mL/min, respectively. Mean indexed eGFR declined from 125.4 to 121.1 mL/min/1.73 m^2^ in the GH group (change of −4.2 mL/min/1.73 m^2^) and from 107.4 to 106.9 mL/min/1.73 m^2^ in the non-GH group (change of −0.5 mL/min/1.73 m^2^). The REML mixed-effects model showed a Group × Time interaction for absolute eGFR (−9.29 mL/min; 95% CI, −13.93 to −4.65; *p* < 0.001) and for indexed eGFR (−3.68 mL/min/1.73 m^2^; 95% CI, −7.34 to −0.03; *p* = 0.048) (Table 4). The age-adjusted sensitivity model showed a Group × Time interaction for absolute eGFR (−6.57 mL/min; 95% CI, −13.09 to −0.06; *p* = 0.048), and cluster-bootstrap inference yielded a percentile 95% CI of −13.59 to −0.57 (*p* = 0.010). The corresponding age-adjusted interaction for indexed eGFR was −3.99 mL/min/1.73 m^2^ (95% CI, −9.15 to 1.16; *p* = 0.129; bootstrap 95% CI, −9.17 to 0.53; *p* = 0.090). For indexed eGFR, bootstrap inference resulted in a percentile 95% CI of, −7.53 to 0.03; (*p* = 0.052) (Table 4). Relative DiD (difference in percent change from baseline) was −3.10% for absolute and −2.88% for indexed eGFR (Table 4).

**Figure 1:**
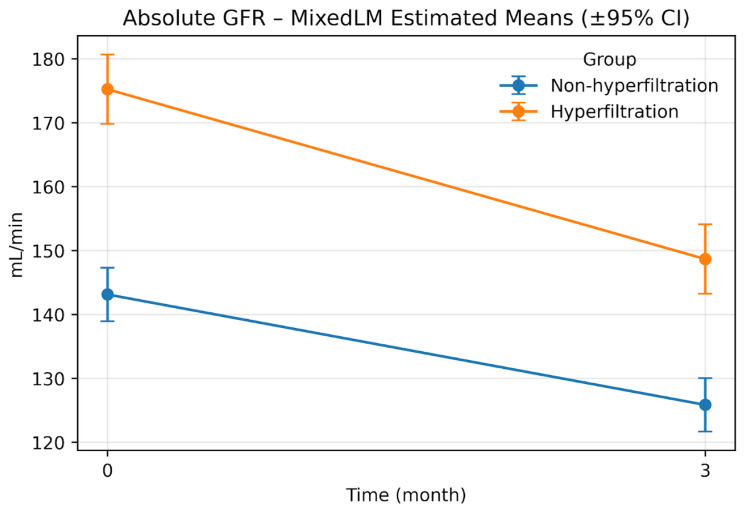
Absolute eGFR at Month 0 and Month 3 by hyperfiltration status. Abbreviations: GFR—glomerular filtration rate.

**Figure 2:**
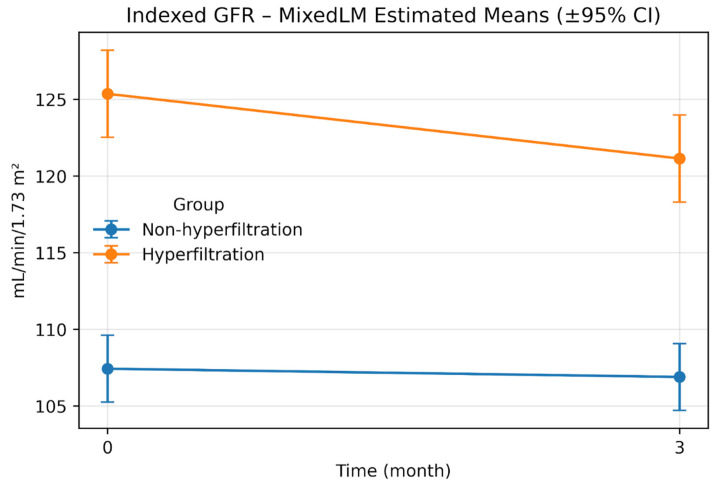
Indexed eGFR at Month 0 and Month 3 by hyperfiltration status. Abbreviations: GFR—glomerular filtration rate.

Residual normality assessments (the Shapiro–Wilk and Anderson–Darling tests) are reported in Supplementary Table S1. In sensitivity analyses, the Group × Time coefficient for indexed eGFR was β = −0.029 (95% CI, −0.065 to 0.006; *p* = 0.105) on the log scale and β = −1.0 × 10^6^ (95% CI, −1.72 × 10^6^ to −2.84 × 10^5^; *p* = 0.006) under the Box–Cox transformation (Supplementary Table S1). On the original scale, the Box–Cox back-transformed difference-in-differences estimate was −3.64 (95% range, −30.58 to 23.76) (Supplementary Table S2). Figure 1 and Figure 2 display the model-estimated trajectories on the absolute and indexed scales.

## 4. Discussion

In this retrospective cohort study of patients undergoing sleeve gastrectomy, those with baseline glomerular hyperfiltration exhibited a significantly larger early decline in absolute eGFR compared to those without. In contrast, changes in indexed eGFR were smaller and less robust. Absolute eGFR thus appears to better capture early postoperative trajectories, which may reflect indexing-related artifacts alongside a physiological alleviation of obesity-related glomerular hemodynamic stress. However, structural injury cannot be excluded without measured GFR, cystatin C, albuminuria, or formal adjudication of postoperative AKI.

The mean difference-in-differences (DiD) in the primary outcome was approximately 9 mL/min, corresponding to an additional ~3.1 percentage-point decline from baseline in the hyperfiltration group relative to the non-hyperfiltration group. This is a small absolute difference and is physiologically compatible with early hemodynamic change. A hemodynamic explanation is plausible, especially because measured-GFR studies after bariatric surgery have described early declines in filtration, with larger changes among patients starting from higher baseline filtration [15,16,17]. Even so, the present data do not establish mechanism. Without measured GFR, cystatin C, albuminuria, or formal AKI adjudication, we cannot distinguish physiological normalization of filtration pressure from altered creatinine generation, perioperative AKI, regression to the mean, or a combination of these processes. The observed between-group difference on the absolute scale should therefore be interpreted as an early postoperative signal in creatinine-based estimation rather than as direct evidence of structural renal benefit or injury.

The divergence between a larger decline on the absolute scale and a minimal change on the indexed scale highlights a size-dependent artifact. This artifact effectively dampens the apparent decline on the indexed scale. Because higher baseline filtration yields larger absolute declines for a given hemodynamic shift, the indexed scale can obscure clinically meaningful changes. Consequently, relying exclusively on indexed eGFR risks underestimating true functional loss and misclassifying patients across drug-dosing thresholds. Our findings align with the methodological cautions articulated in KDIGO 2024, the FDA’s March 2024 Final Guidance, and the FDA ADEPT 8 communication, emphasizing individualized (non-indexed) eGFR for drug dosing at body-size extremes [9,10,23]. Crucially, a recent pooled analysis by Friedman et al. [31] validated this size-dependent artifact using measured GFR (mGFR). They demonstrated that standard BSA-indexed eGFR equations significantly underestimated postoperative GFR reductions, and that de-indexing markedly improved the equations’ accuracy and precision. Moreover, estimating equations poorly predict true filtration changes in individuals with preserved or elevated baseline kidney function, rendering our hyperfiltration cohort particularly susceptible to these artifacts [31].

Our results are parallel to patterns reported in studies using mGFR. Friedman et al. [15] reported a mean postoperative decline of ~17 mL/min; Lieske et al. described a decrease over the first postoperative year in women; and Clerté et al. reported a 6-month decline in mGFR, particularly among individuals with baseline hyperfiltration. These observations have been interpreted as early hemodynamic normalization (glomerular unloading) in those starting from hyperfiltration [15,16,17]. Despite variable mean changes across mGFR studies, the marked decline in our hyperfiltration subgroup aligns with this physiologic rationale without directly proving the mechanism. Our observations align with a recent mGFR-based pooled analysis by Friedman et al. [31], which found a strong correlation between pre-surgery mGFR and the magnitude of postoperative GFR decline. In their study, patients with preserved kidney function experienced substantial mGFR decreases that correlated only weakly with weight loss. This reinforces our premise that early eGFR declines predominantly reflect a physiological reversal of hyperfiltration rather than an artifact of weight loss. Moreover, postoperative mGFR declines independently correlate with reduced systolic blood pressure and HbA1c, suggesting that these filtration shifts reflect the relief of systemic hemodynamic and metabolic stress [31].

A frequent concern with creatinine-based eGFR after bariatric surgery is that postoperative loss of muscle mass reduces creatinine generation, which can artificially elevate eGFR estimates (or mask a decline). That concern remains relevant here. Although absolute eGFR declined in the GH group despite this potential bias, the relative contributions of true filtration change and altered creatinine generation could not be quantified without measured GFR or cystatin C. As recently established by Friedman et al. [31], deindexed, combined creatinine–cystatin C equations yield the highest accuracy and lowest bias compared to mGFR in bariatric patients [31]. Studies that incorporate measured GFR and cystatin C-based equations will be better positioned to separate filtration physiology from changes in creatinine production.

Several limitations warrant consideration. The study design was retrospective, single-center, and limited to a short (3month) follow-up, so the durability and clinical meaning of these early changes are unknown. Analyses relied on creatinine-based eGFR during rapid body-composition change, and postoperative AKI events were not formally adjudicated. We also lacked measured GFR, cystatin C, albuminuria, and detailed medication data, all of which could refine mechanistic attribution and clinical interpretability in future work. Furthermore, the study had no non-surgical control group, so temporal variation and perioperative factors cannot be separated from surgery-associated change. Because we defined hyperfiltration using a baseline indexed eGFR threshold, regression to the mean may contribute to the observed differential change and cannot be fully excluded. Additionally, despite targeted age adjustment, residual confounding from the substantial baseline age difference between groups cannot be fully excluded.

These findings reinforce the KDIGO 2024 Clinical Practice Guideline and the FDA 2024 Final Guidance, which advise against relying solely on BSA-indexed eGFR for drug dosing in patients at extremes of body size [9,10]. Laboratory reporting systems may also consider dual-scale reporting (providing both indexed and absolute values) for patients with BMI > 35 kg/m^2^ to reduce the risk of dosing errors. As non-surgical weight loss modalities, such as modern anti-obesity pharmacotherapy, become increasingly common, these size-dependent indexing artifacts and dual-scale reporting considerations will likely extend beyond bariatric surgery to broader medical weight management.

## 5. Conclusions

Within three months after sleeve gastrectomy, participants with higher baseline indexed filtration exhibited a larger decline in absolute eGFR but only a small change in indexed eGFR. These results show that early postoperative creatinine-based eGFR trajectories are scale dependent. Specifically, absolute eGFR appears to more sensitively capture these early functional shifts, likely reflecting a combination of size-related indexing artifacts and the physiological alleviation of obesity-associated glomerular hemodynamic stress. Because postoperative AKI was not adjudicated and direct kidney function markers were unavailable, this study does not definitively distinguish such physiological hemodynamic adjustment from structural kidney injury. Reporting both absolute (mL/min) and indexed (mL/min/1.73 m^2^) eGFR may aid early postoperative interpretation and help align dosing decisions with rapid postoperative changes in body size.

## Figures and Tables

**Table 1:** Baseline characteristics at Month 0 by hyperfiltration status (mean ± SD or *n* (%)).

Characteristic	Non-Hyperfiltration (*n* = 91)	Hyperfiltration (*n* = 54)	Total (*n* = 145)
Age (years)	41.26 ± 8.54	26.56 ± 4.21	35.79 ± 10.15
Female, *n* (%)	68 (74.7)	41 (75.9)	109 (75.2)
Weight (kg)	115.65 ± 16.61	126.13 ± 27.37	119.55 ± 21.77
BMI (kg/m^2^)	41.84 ± 5.38	43.59 ± 6.80	42.49 ± 5.98
Body surface area (m^2^)	2.30 ± 0.21	2.42 ± 0.29	2.35 ± 0.24
Systolic BP (mmHg)	129.42 ± 15.07	124.63 ± 10.72	127.63 ± 13.77
Diastolic BP (mmHg)	84.59 ± 10.67	80.28 ± 9.08	82.99 ± 10.29
Serum creatinine (mg/dL)	0.74 ± 0.11	0.67 ± 0.09	0.71 ± 0.11
BUN (mg/dL)	12.20 ± 3.16	11.02 ± 2.62	11.76 ± 3.02
Sodium (mmol/L)	139.15 ± 1.95	138.89 ± 1.33	139.06 ± 1.75
Potassium (mmol/L)	4.32 ± 0.33	4.36 ± 0.35	4.33 ± 0.34
Calcium (mg/dL)	9.18 ± 0.36	9.28 ± 0.42	9.22 ± 0.39
Indexed eGFR (mL/min/1.73 m^2^)	107.43 ± 10.16	125.35 ± 4.45	114.10 ± 12.14
Absolute eGFR (mL/min)	143.12 ± 18.77	175.21 ± 23.11	155.07 ± 25.68

Values are mean ± SD or *n* (%). *Abbrevations:* SD—standard deviation; BMI—body mass index; BP—blood pressure; BUN—blood urea nitrogen; eGFR—estimated glomerular filtration rate.

**Table 2:** Anthropometric change from Month 0 to Month 3 by hyperfiltration status (mean ± SD).

Measure	Group	Month 0	Month 3	Δ (Month 3 − Month 0)
Body surface area (m^2^)	Non-hyperfiltration (*n* = 91)	2.30 ± 0.21	2.04 ± 0.17	−0.27 ± 0.07
	Hyperfiltration (*n* = 54)	2.42 ± 0.29	2.12 ± 0.24	−0.30 ± 0.08
	Total (*n* = 145)	2.35 ± 0.24	2.07 ± 0.20	−0.28 ± 0.08

**Table 3:** Estimated marginal means of absolute and indexed eGFR at Month 0 and Month 3 (REML mixed-effects model).

Measure	Group	Month 0 Mean	Month 3 Mean	Δ (Month 3 − Month 0)
Absolute eGFR (mL/min)	Non-hyperfiltration	143.12	125.86	−17.26
	Hyperfiltration	175.21	148.66	−26.55
Indexed eGFR (mL/min/1.73 m^2^)	Non-hyperfiltration	107.43	106.89	−0.54
	Hyperfiltration	125.35	121.14	−4.22

*Abbreviations:* eGFR—estimated glomerular filtration rate; REML—restricted maximum likelihood.

**Table 4:** Between-group contrasts and Group × Time interaction (difference-in-differences) from the REML mixed-effects model.

Measure	Model	Contrast	Parametric β (SE)	95% CI (Wald)	*p*	Bootstrap 95% CI (Percentile)	*p*_Sign
Absolute eGFR (mL/min)	Primary	Hyperfiltration − Non-hyperfiltration (Month 0)	+32.10 (3.49)	[25.25, 38.94]	<0.001	[26.14, 37.88]	<0.001
		Hyperfiltration − Non-hyperfiltration (Month 3)	+22.80 (3.49)	[15.96, 29.65]	<0.001	[17.16, 28.37]	<0.001
		Group × Time (DiD)	−9.29 (2.37)	[−13.93, −4.65]	<0.001	[−14.21, −4.41]	<0.001
		Relative DiD (% of baseline)	−3.10%	—	—	[−5.91%, −0.37%]	0.025
	Age-adjusted	Group × Time (DiD)	−6.57 (3.32)	[−13.09, −0.06]	0.048	[−13.59, −0.57]	0.010
		Relative DiD (% of baseline)	−2.94%	—	—	[−7.04%, +0.60%]	0.100
Indexed eGFR (mL/min/1.73 m^2^)	Primary	Hyperfiltration − Non-hyperfiltration (Month 0)	+17.93 (1.83)	[14.35, 21.51]	<0.001	[15.77, 20.06]	<0.001
		Hyperfiltration − Non-hyperfiltration (Month 3)	+14.24 (1.83)	[10.67, 17.82]	<0.001	[10.88, 17.51]	<0.001
		Group × Time (DiD)	−3.68 (1.87)	[−7.34, −0.03]	0.048	[−7.53, 0.03]	0.052
		Relative DiD (% of baseline)	−2.88%	—	—	[−5.95%, 0.17%]	0.065
	Age-adjusted	Group × Time (DiD)	−3.99 (2.63)	[−9.15, +1.16]	0.129	[−9.17, +0.53]	0.090
		Relative DiD (% of baseline)	−3.31%	—	—	[−7.80%, +0.55%]	0.110

*Abbreviations:* CI—confidence interval; DiD—difference-in-differences; *p*_sign—sign-probability *p* value.

## Data Availability

The datasets generated and/or analyzed during the current study are not publicly available due to patient privacy and institutional data protection regulations but are available from the corresponding author upon reasonable request.

## Supplementary Materials

The following supporting information can be downloaded at: https://www.mdpi.com/article/10.3390/jcm15083001/s1, Table S1. Sensitivity analyses for indexed eGFR on transformed scales; Table S2. Box–Cox smearing back-transformation (indexed eGFR; 10,000 simulations).

## Abbreviations

The following abbreviations are used in this manuscript:

AKI	Acute kidney injury
BMI	Body mass index
BSA	Body surface area
CKD-EPI	Chronic Kidney Disease Epidemiology Collaboration
DiD	Difference-in-differences
eGFR	Estimated glomerular filtration rate
GH	Glomerular hyperfiltration
KDIGO	Kidney Disease: Improving Global Outcomes
mGFR	Measured glomerular filtration rate
