# Supplementary material for: Body Surface Area Indexing Attenuates Apparent Early eGFR Decline After Sleeve Gastrectomy: A Retrospective Cohort Study
Source: J Clin Med. 2026 Apr 15;15(8):3001. doi: 10.3390/jcm15083001 (PMC13116381; doi:10.3390/jcm15083001)
Supplement: Supplementary file 1 [file jcm-15-03001-s001.zip › Supplementary Tables.pdf]

Supplementary Table S1. Sensitivity analyses for indexed eGFR on transformed scales

**Scale**	**Group×Time β**	**95% CI (Wald)**	***p***	**Residual normality**
log(Indexed eGFR)	−0.029	[−0.065, +0.006]	0.105	Shapiro/AD: deviation
Box–Cox (λ optimized)	-1.0×10^{6}	$[-1.72\times10^{6}, -2.84\times10^{5}]$	0.006	Shapiro/AD: deviation

Shown are Group × Time coefficients (β) with Wald 95% CIs and two-sided p values. Residual normality summaries are based on the Shapiro–Wilk and Anderson–Darling tests. For the Box–Cox model, λ was optimized by maximum likelihood. Because Box–Cox transformations rescale the outcome, β coefficients on the transformed scale are not directly interpretable in original eGFR units; inference is therefore emphasized on the original scale and via bootstrap uncertainty.

Supplementary Table S2. Box–Cox smearing back-transformation (indexed eGFR; 10,000 simulations)

Group	Time	Mean (mL/min/1.73 m²)	95% range (mL/min/1.73 m²)
Non-hyperfiltration	Month 0	107.89	[89.61, 121.24]
Non-hyperfiltration	Month 3	108.17	[90.30, 121.69]
Hyperfiltration	Month 0	125.34	[114.01, 134.99]
Hyperfiltration	Month 3	121.82	[109.48, 132.13]

Contrast	Estimate	95% range
Δ Non (3–0)	+0.08	[−22.18, 22.31]
Δ Hyper (3–0)	−3.56	[−19.14, 11.53]
DiD (Hyper − Non)	−3.64	[−30.58, 23.76]

Means are back-transformed to original units (mL/min/1.73 m²). Intervals denote simulation-based 95% intervals. ***Abbreviations:*** DiD, difference-in-differences.
